# Pathogenesis-Related Gene Expression in Response to *Trachyspermum ammi* Supplementation Along With Probiotics in Chicken Salmonellosis and Insights in Drug Therapeutics

**DOI:** 10.3389/fvets.2022.866614

**Published:** 2022-06-02

**Authors:** Zulfqarul Haq, Syed Mudasir Ahmad, Ishrat Bashir, Mashooq Ahmad Dar, Afnan Saleem, Azmat Alam Khan, Mohammad Iqbal Yatoo, Shabir Mir, Ankur Rastogi, Mohd Isfaqul Hussain, Riaz A. Shah, Basharat Bhat

**Affiliations:** ^1^Division of Livestock Production and Management, Faculty of Veterinary Sciences & Animal Husbandry, Sher-e-Kashmir University of Agricultural Sciences and Technology of Kashmir, Srinagar, India; ^2^Division of Animal Biotechnology, FVSc & AH, Shuhama, Sher-e-Kashmir University of Agricultural Sciences and Technology of Kashmir, Srinagar, India; ^3^Department of Clinical Biochemistry, University of Kashmir, Srinagar, India; ^4^Division of Veterinary Microbiology, Faculty of Veterinary Sciences and Animal Husbandry, Shuhama, Sher-e-Kashmir University of Agricultural Sciences and Technology of Kashmir, Srinagar, India; ^5^Division of Animal Genetics and Breeding, FVSc & AH, Shuhama, Sher-e-Kashmir University of Agricultural Sciences and Technology of Kashmir, Srinagar, India; ^6^Division of Animal Nutrition, Faculty of Veterinary Sciences and Animal Husbandry, Sher-e-Kashmir University of Agricultural Sciences and Technology of Kashmir, Srinagar, India

**Keywords:** *Salmonella* infection, chicken, dietary plant extracts, gene expression, antioxidant activity (AA), drug therapeutics

## Abstract

*Salmonella enterica* serovar *typhimurium* (*S. typhimurium*) is the leading cause of foodborne illness. Since *Salmonella* continues to have a detrimental effect on public health, there is an ongoing need to develop more advanced methods for combating Salmonellosis in foods before they reach consumers. In addition, the quest for alternative natural products has recently intensified due to increasingly stringent regulations regarding the use of antibiotics as growth promoters and consumer demand for antibiotic-free poultry products. This study evaluated the effect of Ajwain extract (AJE) on immune response and antioxidant status in broiler chicks challenged with *Salmonella typhimurium*. The chicks were infected with *S. typhimurium* and were divided into the different groups, except for the control group (CON). The challenged chicks received different treatments with 3 × 10^9^ colony-forming unit (CFU) Acipro^TM^-WS probiotic (PRO), 200 mg/kg Ajwain extract (AJE), 200 mg/100 kg of enrofloxacin (ENR), and a combination of 3 × 10^9^ CFU Acipro^TM^-WS probiotic and 200 mg/kg Ajwain extract (COM). Five days posttreatment, the tissue samples (liver and spleen) were analyzed. The results showed that basal diet supplemented with Ajwain extract (AJE) and a combination of probiotic and Ajwain extract (COM) significantly (*P* < 0.0.5) reduced the cytokine expression in broiler chicks challenged with *S. typhimurium*. Our findings suggest that AJE can clear the bacterial infection, improve antioxidant status, and suppress the inflammation response. Additionally, AJE supplementation significantly mitigated the *S. typhimurium*-induced increase in the interleukin-6 (IL-6) (liver and spleen), interleukin-8 (IL-8) (liver and spleen), interleukin-17A (IL-17A) (liver and spleen), and inducible nitric oxide (iNOS) (spleen and liver) levels (*P* < 0.05). We conclude that Ajwain is an efficient feed additive with antioxidant and anti-inflammatory properties. The interaction networks developed in this study provide a novel lead that could be targeted for anti-inflammatory and antioxidant properties.

## Introduction

*Salmonella enterica* is a facultative intracellular pathogen capable of causing disease in a broad host range. Especially, *Salmonella enterica* serovar *typhimurium* is one of the most common pathogenic bacteria, which is known to cause severe economic losses to the poultry sector. *Salmonella* is considered as a severe foodborne pathogen that leads to about 1.2 million illnesses, 23,000 hospitalizations, and around 450 deaths per year in the United States of America (1). Poultry and eggs are considered as one of the most important reservoirs, transmitted to humans through consumption of infected animals or animal products or direct contact with infected animals or through the fecal-oral route (2). Most birds infected by *Salmonella typhimurium* (*S. typhimurium*) do not show clinical signs and remain asymptomatic. However, the clinical disease in terms of reduced growth, loss of egg production, and mortality has been observed in birds exposed to many stress conditions or in young broiler chicks with an immature immune system. *Salmonella typhimurium* has been shown to survive and replicate within avian macrophages, which is essential for the full expression of its virulence. In addition, a distinct array of cytokines that are released in response to *Salmonella* infection contributes to the development of inflammatory reactions.

Vaccination, sanitation, and antibiotics are the most common methods used to combat *Salmonella* infections. As the vaccination against *Salmonella* is not always practical, antibiotics are preferred. The major issues associated with widespread antibiotic use are the emergence of antibiotic-resistant bacteria and the accumulation of antibiotic residues in food for human consumption (3, 4). The WHO and the Centers for Disease Control and Prevention (CDC) have declared rapid growth of antimicrobial resistance (AMR) as one of the major global health concerns and a rising threat of the 21st century (5). According to reports, by the year 2050, AMR would be responsible for an estimated 10 million fatalities per year worldwide, with a medical cost of up to USD 100 trillion (6). Study and the poultry industry are in desperate need of effective and long-lasting alternatives to antibiotics because of increased demand from the WHO/EU to minimize the use of antibiotics as feed additives and growth promoters (7). In the recent years, several feed additives have been suggested to replace antibiotic growth promoters such as probiotics, prebiotics, synbiotics, essential oils, acidifiers, enzymes, and organic phytochemicals. However, these feed additives have not produced consistent and satisfactory results in terms of growth, feed conversion ratio, and reduced mortality worldwide have resulted in less adaptability at the farm level (8).

In light of the above discussion, it is quite clear that it is more logical to seek out different permits and combinations of organic herbs and other organic alternatives that can effectively reduce the pathogenicity of *Salmonella* and act as a promising alternative to antibiotic growth promoters in poultry. The addition of such additives to feed poultry may have several beneficial effects, including the rapid development of healthy gut microflora and stabilization of digestion and improved feed efficiency, reduction of bacterial load, improved immune responses, and antioxidant status. In broiler chicken, probiotics have shown improvement in growth performance, nutrient digestibility, immunity, and intestinal health (9), suggesting the potential value of probiotics as an alternative to antibiotics in chicken farming. Plant feed additives constitute a broad group of biologically active compounds with potentially positive effects on animal health and productivity, which enhance health in a bird's digestive system by reducing the number of diseases causing bacteria (10).

Ajwain (*Trachyspermum ammi*) is an aromatic, annual medicinal herb that belongs to the family Apiaceae and is a highly valued, medicinally important seed spice. It has been reported that Ajwain contains principle active compounds, including phenols, mainly thymol and carvacrol, which are pharmacologically active substances (11) and have antiseptic, antifungal, antibacterial, and anthelminthic effects (12, 13). Ajwain has the potential for use as a source of natural antioxidants and this property is directly related to the number of total phenols and flavonoids. Some researchers had reported an increase in body weight and decrease in feed efficiency when Ajwain and other herbal ingredients were used as a dietary supplement in broiler diets. Ajwain's important role in broiler diets is antibacterial activity and improvement in the immune response (14). These two factors may contribute to better growth and performance in broilers. Therefore, this study was conducted to determine the effects of dietary supplementation of probiotics, Ajwain extract, and a mixture of Ajwain extract and probiotic on the inflammatory response, oxidation status, and bacterial count in broiler chicken challenged with *Salmonella*.

## Materials and Methods

### Plant Material

Ajwain seeds were obtained locally and crude extract material was prepared by the following method. The seeds were dried under room temperature at 30 ± 2°C. After drying the seeds for 3 days, the dried seeds were grinded into powder using mortar and pestle and sieved with a sieve of 0.3 mm aperture size. The powder obtained was successively extracted in ethanol by using Soxhlet extractor (60–80°C). Size of the Soxhlet was 200 ml in which the grinded material was put in the upper part and the extract was obtained in the lower part at specific temperatures of the solvents used. The ethanolic extract was then concentrated with the help of rotary evaporator under reduced pressure and the solid extracts were stored in refrigerator at 4°C for further use.

### 2,2-Diphenyl-1-Picrylhydrazyl Radical Scavenging Assay

Various concentrations of ethanolic plant extract (100–700 μg/ml) were added to 1 ml of the 20 mg/100 ml methanol solution of 2,2-diphenyl-1-picrylhydrazyl (DPPH). The mixture was shaken vigorously and kept at room temperature for 30 min in the dark and the absorbance was measured at 517 nm. Lower absorbance of the reaction mixture indicates higher free radical scavenging activity. Ascorbic acid was taken as known standard positive control. Percentage inhibition activity was calculated by:


$$\begin{array}{l} \%inhibition=\left(A0--A1\right)/A0\times 100 \end{array}$$

Where, A0 was the absorbance of the control and A1 was absorbance in the presence of plant extract/known antioxidant.

### Ferric Reducing Antioxidant Power (FRAP) Assay

This method is based on the reduction of Fe^3+^ 2,4,6-tripyridyl-S-triazine (TPTZ) complex to Fe^2+^-tripyridyltriazine (blue-colored complex) formed by the action of electron donating antioxidants.

Different concentrations of ethanolic extract were mixed with FRAP reagent (300 mM acetate buffer, 10 ml TPTZ in 40 mM HCl, and 20 mM FeCl_3_.6H_2_O). An intense blue color complex was formed and then the absorbance at 593 nm was recorded against a reagent blank. The calibration curve was prepared by plotting the absorbance at 593 nm against different concentrations of FeSO_4_. The FRAP values were obtained by comparing the absorbance change in the test mixture with those obtained from increasing concentrations of Fe^3+^.

### Experimental Design and Animal Management

A 100-day-old Cobb 400 broiler chicks were procured from a local hatchery in Kashmir. Chicks were kept under standard brooding conditions in the animal house at the Faculty of Veterinary Sciences and Animal Husbandry, Shuhama (SKUAST-K), India. The chicks were fed basal diets starting from day zero as per the recommendations of the Indian Council of Agricultural Research (ICAR) 2013. The ingredients and chemical composition (%) of broiler crumb feed are given in Table 1.

**Table 1:** Ingredient and chemical composition (%) of broiler crumbs diets.

**Ingredient**	**Pre-starter**
Maize grain	59
Soybean meal	32.20
Meat-cum-bone meal	4.80
Vegetable oil	2.20
De-oiled rice bran	-
Limestone powder	0.60
Dicalcium phosphate	0.10
Lysine	0.1
Methionine	0.35
Salt	0.35
Choline chloride	0.25
Trace minerals mixture	0.10
Vitamin premix	0.05
**Chemical composition (%DM) of the diets fed to experimental broiler**	
CP	22.02
EE	4.91
Total Ash	5.28
CF	4.10
ME (kcal/kg)^#^	3004.00

### Challenge With *Salmonella typhimurium* and Treatment

After giving an adaptation time of 3 days to the Cobb broiler chicks, all the groups were orally inoculated with [2×10^8^ colony-forming unit (CFU)/ml] at day 4 of age except control that was subjected to a mock challenge with 1 ml of nutrient broth orally. After 12-h postinfection, fecal swabs were taken from all the chicks and incubated overnight in tetrathionate broth at 42°C. The overnight culture was streaked on Brilliant Green Agar (BGA) and MacConkey plates. Following overnight incubation, the colonies on the plates were then examined. *Salmonella typhimurium* strain (ATCC 14028) was procured from the American Type Culture Collection. The culture was revived in tetrathionate broth (TTB) after incubating 37°C for 18 h. The overnight growth from TTB was streaked on MacConkey and Brilliant Green Agar (BGA) plates, incubated at 37°C for 24 h, and finally examined for typical *Salmonella* colonies.

After challenging *Salmonella*, the chicks were randomly allocated into the six treatment groups. The control group (CON) comprised unchallenged birds fed a basal diet and the infected group (INF) was challenged with *Salmonella* infection. The antibiotic group (ENR) was fed a basal diet supplemented with 200 mg/100 kg of enrofloxacin. The probiotic group (PRO) consisted of challenged birds fed a basal diet supplemented with 3 × 10^9^ CFU Acipro^TM^-WS. The Ajwain extract group (AJE) comprised challenged birds fed a basal diet supplemented with 200 mg/kg Ajwain extract. Finally, the combination group (COM) consists of challenged birds fed a basal diet and supplemented with 200 mg/kg Ajwain extract and 3 × 10^9^ probiotic mixture.

### Sample Collection

Following 5 days posttreatment, we took six birds at random from every group and euthanized them with CO_2_. The tissue samples (liver and spleen) were aseptically removed from the chicks. The liver and spleen tissue samples were stored at −80°C until RNA extraction. Blood samples were also taken to assess oxidation status.

The clinical scores were recorded twice daily following a points-based scoring system (15). In addition, the carcass of sacrificed birds was subjected to a systemic necropsy procedure to examine and record the lesions characteristic of Salmonellosis, which predominantly included bronze discoloration of liver, splenomegaly, and typhlitis.

### Bacterial Count Measurement in the Tissue

The bacterial counts were measured in the liver and spleen. The sample collection was done aseptically, weighed, diluted in 3 ml of sterile phosphate-buffered saline (PBS), and then homogenization at 60 Hz for 60 s using the Scientz-48 homogenizer. Finally, 100 μl of the homogenate was plated on Xylose Lysine Deoxycholate (XLD) agar and incubated at 37°C for 24 h. Subsequently, the bacterial counts were calculated as log CFU per gram of tissue.

### Total Antioxidant Status

Total antioxidant status was estimated through a new direct automated method given by Erel (16). 200 μl of reagent 1 (R_1_) was taken in each well of an uncoated plate to which 5 μl of serum and 20 μl of reagent 2 (R_2_) were added and the absorbance was taken at a wavelength of 600 nm.

### Total Oxidant Status

Total oxidant status was estimated through a new automated colorimetric method given by Erel (16). 225 μl of reagent 1 (R_1_) was taken in a well of an uncoated plate, 35 μl of serum and 11 μl of reagent 2 (R_2_) were added to the well, and reading was taken at a wavelength of 560 nm.

### Ribonucleic Acid Extraction and Complementary DNA Synthesis

Total RNA was extracted from the tissue samples (liver, spleen, and cecum) by Trizol™ method (Invitrogen, USA) per the manufacturer's protocol. The quantity and quality of isolated RNA were checked at 260 and 280 nm with UV–visible spectrophotometer. Prior to complementary DNA (cDNA) synthesis, RNA samples were run on 1% agarose gel. DNase treatment using the DNase1 Kit (Sigma, USA) was given to rule out any genomic DNA contamination. cDNA synthesis was done with an equal concentration of RNA (1.5 μg/μl) in all the samples using the Thermo Scientific RevertAid First Strand cDNA Synthesis Kit™ (Lithuania) oligo dT primers following the manufacturer's protocol. For validation of cDNA and primers, conventional PCR was run using cDNA as a template. The primers used for interleukin-6 (IL-6), interleukin-8 (IL-8), interleukin-17A (IL-17A), inducible nitric oxide synthase (iNOS), glyceraldehyde-3-phosphate dehydrogenase (GAPDH), and β-actin gene amplification were taken from previous studies (Table 2) (17, 18).

**Table 2:** List of primers used for quantitative real-time PCR. T_A_ = annealing temperature.

**Gene**	**Primer sequence (5^**′**^ → 3^**′**^)**	**T_**A**_ (**°**C)**	**Amplicon size**	**Reference**
β Actin	Forward primer TGGCATTGCTGACAGGAT Reverse primer CTGCTTGCTGATCCACAT	63	160 bp	(17)
GAPDH	Forward primer GTCAGCAATGCATCGTGCA Reverse primer GGCATGGACAGTGGTCATAAGA	60	180 bp	(18)
IL-6	Forward primer GCTCGCCGGCTTCGA Reverse primer GGTAGGTCTGAAAGGCGAACAG	63	71 bp	(19)
IL-8	Forward primer GGCTTGCTAGGGAAATGA Reverse primer AGCTGACTCTGACTAGGAAACTGT	55	200 bp	(20)
IL-17A	Forward primer TTTCTGCACATGGGAAGGTG Reverse primer CCTGGTTCATGTTGCTGATGC	61	144 bp	(21)
iNOS	Forward primer AAAGAAAGGGATCAAAGGTGGT Reverse primer CAAGCATCCTCTTCAAAGTCTG	60	296 bp	(22)

### Quantitative Real-Time PCR

The messenger RNA (mRNA) expression levels of IL-6, IL-8, IL-17A, and iNOS genes of chicken were determined by real-time PCR (Roche™, Germany), using SYBR green as a fluorescent dye. β-actin and GAPDH were used as an internal control. The relative quantification of IL-6, IL-8, IL-17A, and iNOS mRNA was determined by 2^−ΔΔCT^ method (23), where ΔΔCT corresponded to the difference between the CT measured for the mRNA level of each tissue and the CT measured for the mRNA level of the reference gene, ΔCT = CT (target gene)—mean CT (β-actin) and CT (GAPDH).

### Network Analysis

To identify the possible chemical–gene and gene–disease interaction, we utilized the Comparative Toxicogenomics Database (CTD) (version November 2016) (24) and Drug Bank database (version 5.0), respectively (25). In addition, two topographical features, betweenness and degree, were used to filter binding terms interacting with genes of interest.

## Results

### 2,2-Diphenyl-1-Picrylhydrazyl Radical Scavenging Assay and FRAP Assay

At 700 μg/ml, the ethanolic extract has shown 77.26% of inhibition when compared with the standard ascorbic acid, which has shown 95.75% of inhibition. The inhibition has increased with increase in the concentration of extract. Similarly, at 700 μg/ml, the ethanolic extract showed 3.16% of inhibition and the inhibition increased with the increase in the concentration of the extract (Supplementary Table 1).

### Clinical Signs

**Figure 1:**
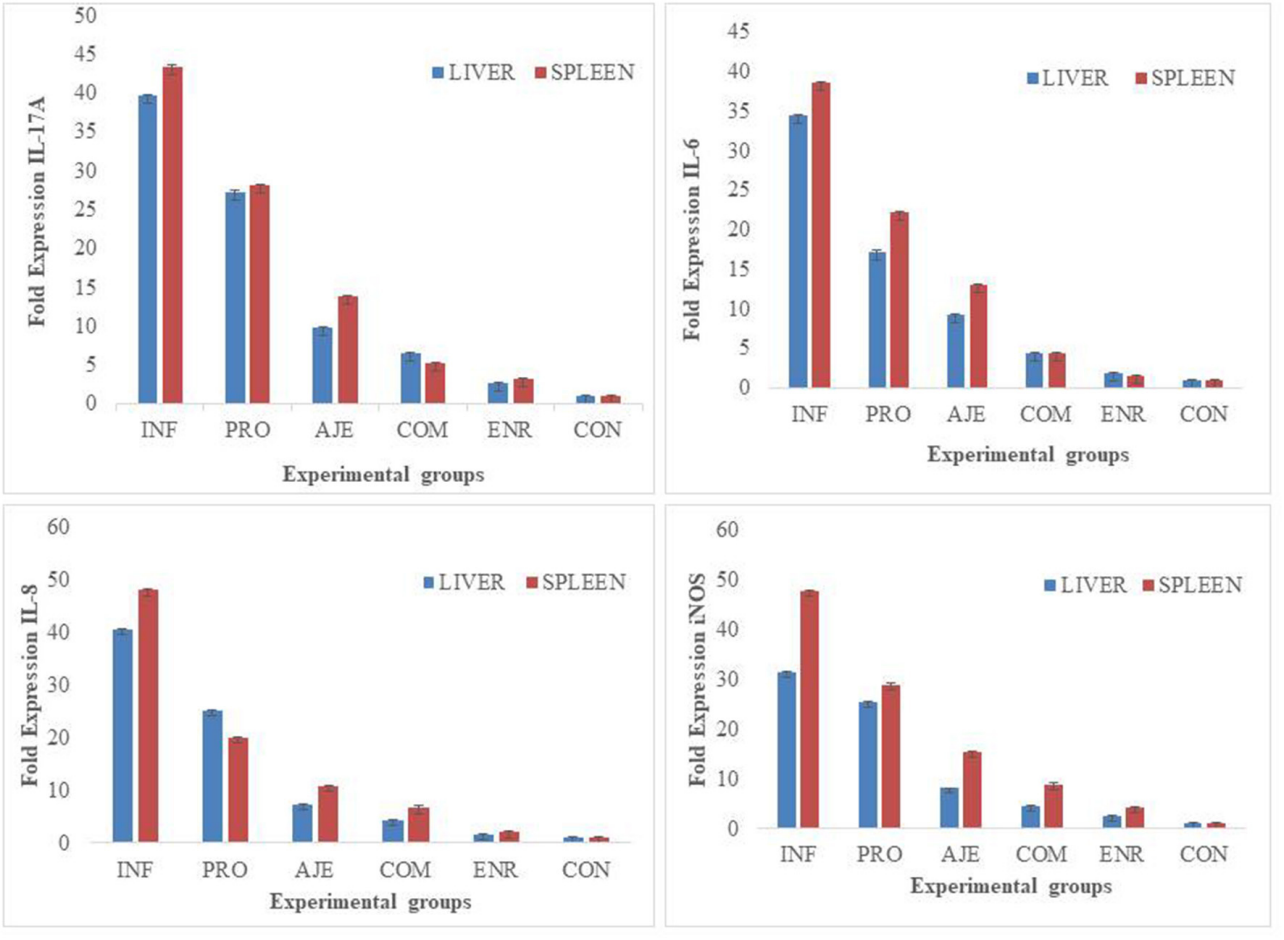
Proportionate distribution of gross lesions in chicks challenged with *Salmonella typhimurium* infection. (INF group) infected with *S. typhimurium*; (CON group) control without any treatment; (AJE group) infected with *S. typhimurium* treated with ajwain extract; (PRO group) infected with *S. typhimurium* treated with probiotic; (COM group) infected with *S. typhimurium* treated with a mixture ajwain extract and probiotic; (ENR group) infected with *S. typhimurium* treated with antibiotic.

Birds showed clinical signs after inoculating the chicks with 2 × 10^8^ CFU/ml of *Salmonella typhimurium*. Depression, dullness, inappetence, and reluctance to move with both the eyes closed were some of the symptoms. The birds showed weakening, anorexia, increased thirst, drooping of wings, ruffled feathers, lowered heads, and diarrhea. Birds treated with the PRO, AJE, COM, and ENR groups had fewer clinical symptoms than the INF group. The control group with the same structure as the challenged groups was kept in a separate room to avoid cross-contamination; therefore, no signs were seen. The proportionate distribution of gross lesions in each group followed the pattern: INF > PRO > AJE > COM > ENR > CON (Figure 1).

### Bacterial Counts in the Tissue

**Figure 2:**
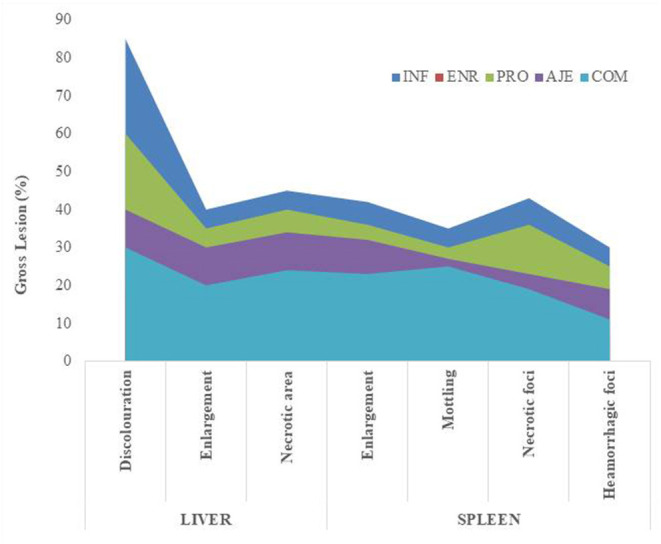
Effect of AJE (ajwain extract), PRO (probiotic), COM (ajwain extract + probiotic) and ENR (antibiotic) on the bacterial counts (log CFU/g) in the spleen and liver of chicks challenged with *Salmonella typhimurium* infection.

After the challenge, no *S. typhimurium* was found in the CON group and very little *S. typhimurium* was found in the ENR group in the liver. Bacterial load was significantly lower (*P* < 0.05) in the AJE- and COM-treated groups compared to the INF group. In spleen, *Salmonella typhimurium* was not detected in the control group and was very low in the ENR group postchallenge. Bacterial counts were significantly higher (*P* < 0.05) in the COM and PRO groups compared to the ENR group. Bacterial count in the ENR group was significantly lower (*P* < 0.05) than in the infected group. The bacterial count was slightly lesser in the AJE-treated group than in the PRO- and COM-treated groups (Figure 2).

### Total Oxidant Status of Broilers Challenged With *Salmonella typhimurium*

There was no significant difference (*P* ≥ 0.05) among various groups for the total oxidant status (TOS) levels, while the control group showed slightly lower TOS levels than the other groups, as shown in Table 3. This may be because of less oxidative stress in the control group compared to the infected and treated groups. Infection induces oxidative stress, which is also evident from the raised inflammatory cytokines/mediators levels in the infected groups compared to the control group.

**Table 3:** Total oxidant status (TOS) of broiler challenged with *Salmonella typhimurium* infection.

**Group**	**Mean ±SEM**
INF	0.0728 ± 0.00272
PRO	0.0739 ± 0.00175
AJE	0.0781 ± 0.00137
COM	0.0748 ± 0.00044
ENR	0.0757 ± 0.00342
CON	0.0722 ± 0.00037

### Total Antioxidant Capacity of Broiler Challenged With *Salmonella typhimurium*

The probiotic- and antibiotic-treated groups showed significantly (*P* < 0.05) lower TAS than control; however, levels were higher in the Ajwain-treated group, as shown in Table 4. Rest of all showed non-significant differences (*P* ≥ 0.05). This may be due to the antioxidant nature of Ajwain.

**Table 4:** Total antioxidant capacity (TAS) of broiler challenged with *Salmonella typhimurium* infection.

**Group**	**Mean ±SEM**
INF	
PRO	0.9455 ± 0.09679
AJE	1.0912 ± 0.17768
COM	
ENR	0.9382 ± 0.01209
CON	1.4686 ± 0.19206

### Gene Expression Study

**Figure 3:**
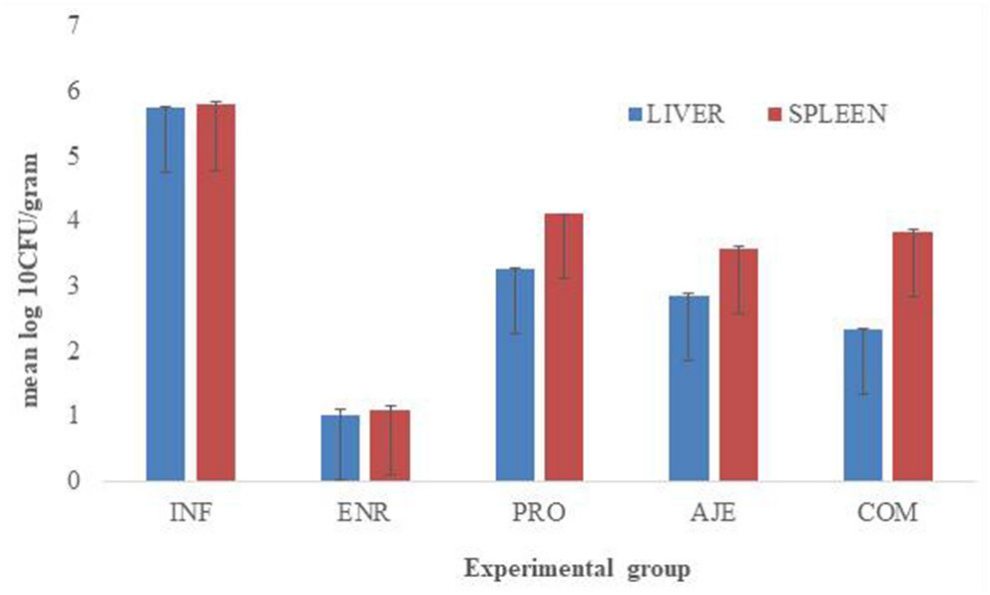
Expression of IL-6, IL-8, IL-17A and iNOS in liver and spleen of broilers fed diets and supplemented with different treatments after *Salmonella typhimurium* infection on day 5PI. (INF group) infected with *S. typhimurium*; (CON group) control without any treatment; (AJE group) infected with *S. typhimurium* treated with ajwain extract; (PRO group) infected with *S. typhimurium* treated with probiotic; (COM group) infected with *S. typhimurium* treated with a mixture ajwain extract and probiotic; (ENR group) infected with *S. typhimurium* treated with antibiotic. Each bar represents the standard error.

Quantitative real-time PCR revealed significant cytokine expression levels in the treated birds in both the liver and spleen compared to the birds of the INF group (Figure 3). The expression of IL-6, IL-8, IL-17A, and iNOS was significantly (*P* < 0.05) downregulated in bird's diet supplemented with the AJE and COM groups compared to the PRO and INF groups. Expression was significantly (*P* < 0.05) more significant for the INF group than the CON or ENR group. The ENR group was upregulated but not statistically significant (*P* < 0.05) than the CON group. The expression of IL-6 and IL-8 in both the liver and spleen in all the supplemented groups (PRO, AJE, and COM) was significantly downregulated (*P* < 0.05) than IL-17A and iNOS. All the differentially expressed genes such as IL-6, IL-8, IL-17A, and iNOS were expressed more in the spleen than the liver.

### Network Analysis

**Figure 4:**
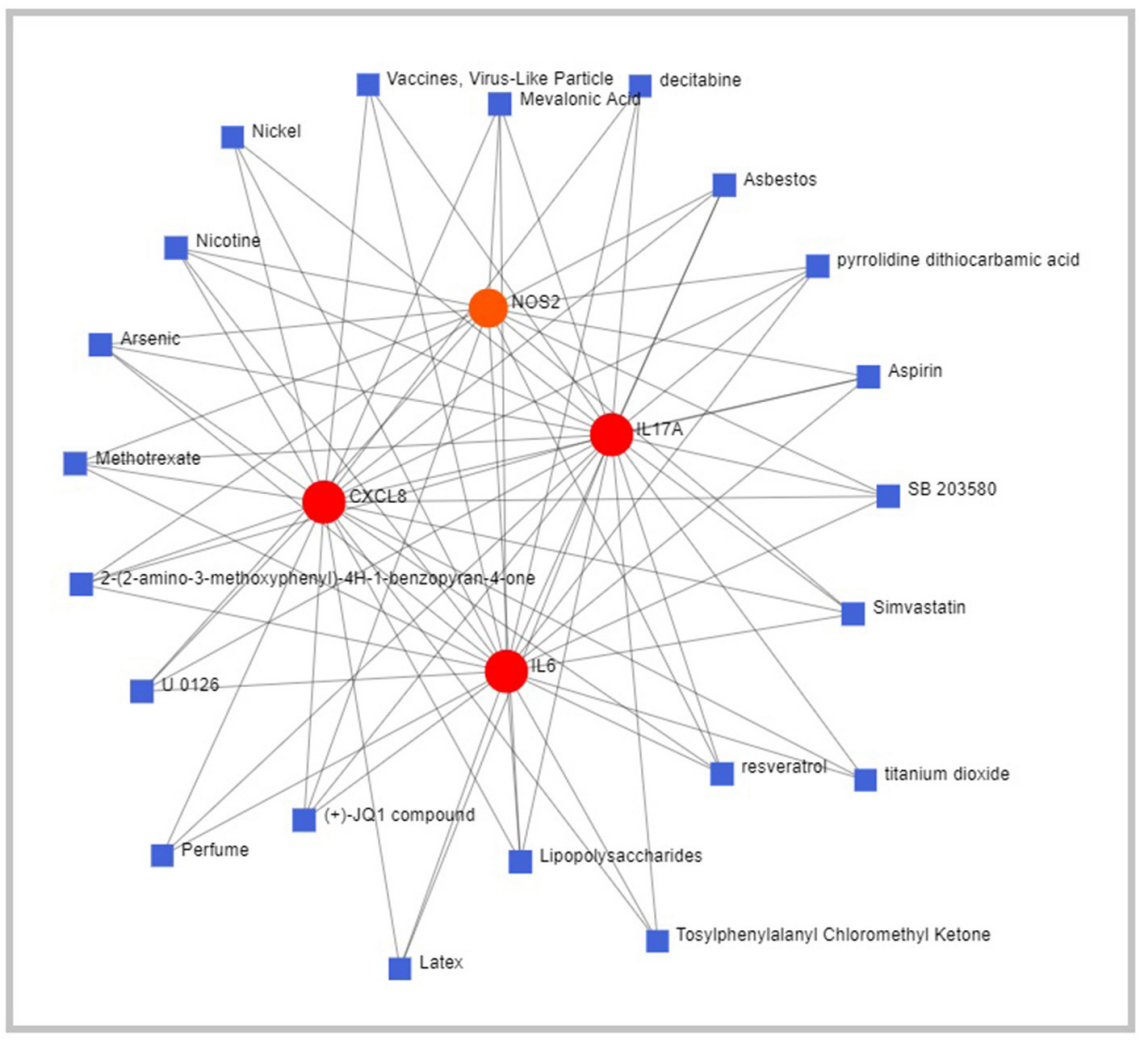
Schematic diagram of Network and Pathway Analysis of Chemical-Gene interaction using Comparative Toxicogenomics Database (CTD) (Version Nov. 2016).

**Figure 5:**
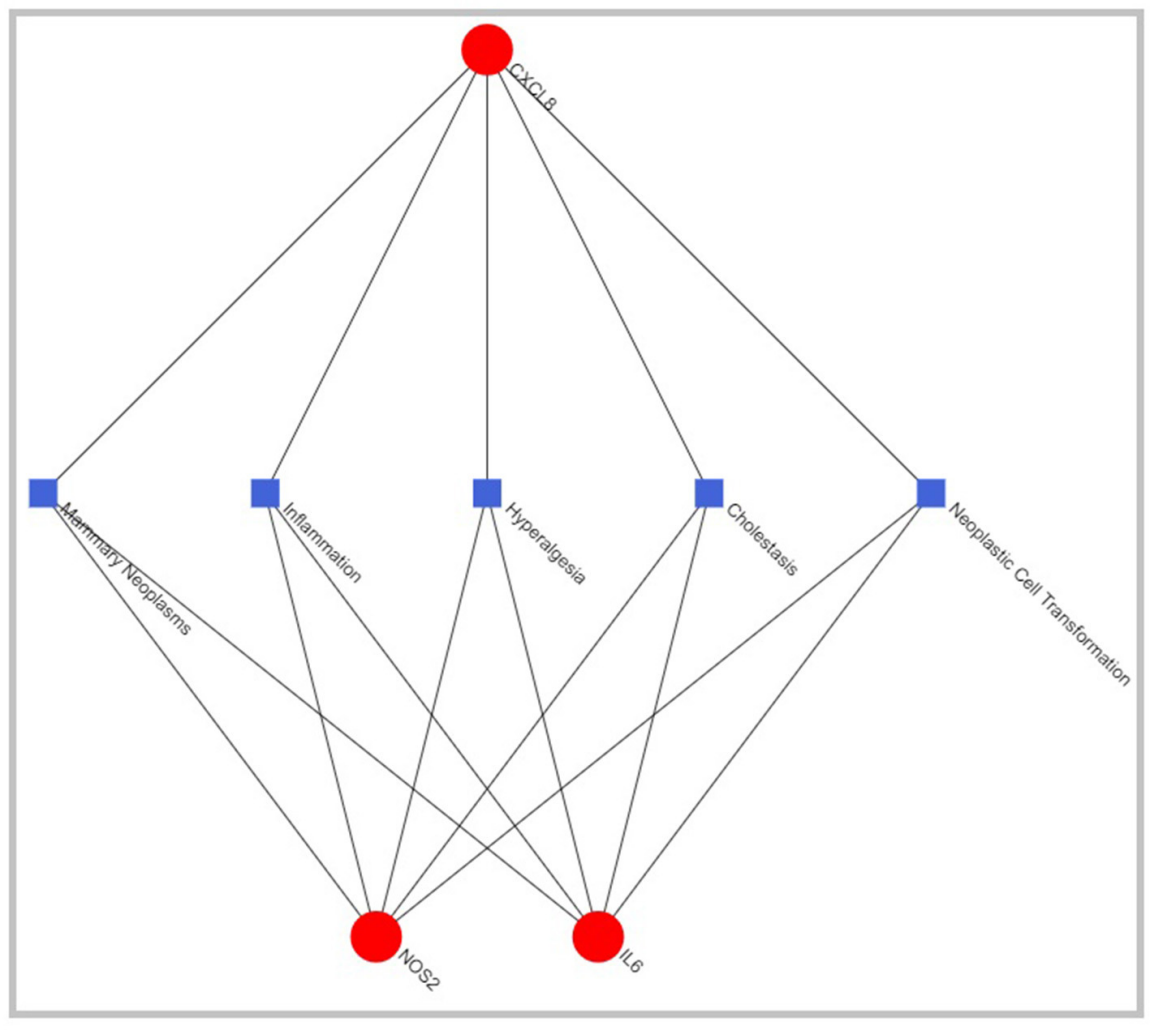
Schematic diagram of Gene Disease interaction Network.

Chemical–gene interaction network suggests that C-X-C motif chemokine ligand 8 (CXCL8), IL-6, IL-17A, and Nitric oxide synthase 2 (NOS2) interacts with small molecules such as asbestos, pyrrolidine dithiocarbamate (PDTC), aspirin, simvastatin, titanium oxide, resveratrol, lipopolysaccharide (LPS), latex protein (LP) fraction, methotrexate, arsenic, nicotine, and nickel chloride (Figure 4). The gene-disease interaction network suggests the potential role of CXCL8, NOS2, and IL-6 in mediating mammary neoplasms, inflammation, hyperalgesia, cholestasis, and neoplastic cell transformation (Figure 5).

## Discussion

*Salmonella* infections in poultry flocks can cause acute and chronic clinical diseases and have received greater international attention in the recent years because of their role in foodborne outbreaks of human illness. *Salmonella* has been transmitted horizontally through the respiratory tract or the digestive tract by fecal matter contaminants or vectors in chicken. It can also be transmitted vertically through the egg from the diseased breeders to the offspring. Several reports have pointed out that contaminated poultry and their products are the primary sources of human infection with *Salmonella*, which mainly colonizes the intestine, liver, and spleen (26, 27). Due to public health and flock health concerns, *Salmonella* infections cause economically significant losses for poultry producers.

Moreover, in the poultry industry, there is a clear demand for safer alternatives for feed additives as a result of the ban on using in-feed antibiotics in the European Union. Several studies have examined the ability of medicinal plants and their essential oils or extracts to promote growth in broiler chickens (28, 29). In this respect, Ajwain is an herb long known for its medicinal properties (30). Several medicinal properties of Ajwain seeds have been demonstrated in broiler chickens, including antibacterial, antioxidant, and antifungal properties (31). The most active compounds in Ajwain are thymol and carvacrol, which are important pharmacologically active substances. In addition, the major compounds reported include γ-terpinene (31.02%), thymol (29.20%), p-cymene (22.87%), and β-pinene (4.13%) (32).

In this study, clinical symptoms were evident time dependent after inoculating the birds with *S. typhimurium*. Infected birds showed signs of exhaustion, marked depression, progressive lassitude and somnolence with closed eyes, shivering, huddling and reluctance to move, whitish diarrhea, high mortality, weight loss, lameness, respiratory manifestations, and pasty diarrhea (33). The severity of symptoms was reduced in the other treated groups compared to the infected group. The broiler chicks fed with diets containing probiotics, Ajwain extract, and antibiotics prior to challenge significantly reduced the symptoms compared to the infected group.

This study was conducted to investigate the effect of Ajwain extract supplementation on the inflammatory immune activities, antioxidant capacity, and bacterial load in the spleen and liver during immunological and oxidative stress induced by *Salmonella typhimurium*. Researchers have recently been trying to find novel feed additives using natural sources, including plant materials, to prevent and treat oxidative stress in livestock (34, 35). By this study, supplementation of Ajwain extract and a mixture of probiotic and Ajwain to the diets of broiler chicks significantly improved their performance (36). This study revealed that Ajwain extract supplementation lowered cytokines and enhanced oxidative stress. Previous studies showed that the alcoholic extract of Ajwain has flavonoids, saponins, and tannins (37). Ajwain seeds contain essential oil (2–3%), containing about 40–50% thymol. Other ingredients are rho-cymene, beta-pinene, dipentene, β-terpinene, and carvacrol and they are well-known for antibacterial, antihelminthic, and antioxidant effects (38). This study revealed that the treatment group fed with Ajwain and a mixture of Ajwain and probiotic showed significantly (*P* < 0.05) reduced bacterial count in both the liver and spleen compared to the other treatment groups, but were higher than that the antibiotic group. Ajwain essential oils can inhibit the growth of bacteria associated with gastrointestinal infections such as *Escherichia coli* and *Salmonella typhimurium* (36, 39). The diets supplemented with the antibiotic group prior to the challenge significantly reduced the number of mortalities resulting from infection with *Salmonella*. The chicks fed with basal diet and supplemented with probiotics restricted the colonization by *Salmonella* and helped to develop intestinal architecture and improving the host immunity, which significantly contribute to the nutrient absorption. Probiotics are essential modulators in the intestine and facilitate host immunity by releasing bioactive compounds such as mucins, chemokines, cytokines, and defensins (40).

The increased total oxidant status of the challenged group (INF) compared to the control group might be due to infection and raised levels of inflammatory cytokines. Ajwain supplementation has been found to significantly enhance the activity of antioxidant enzymes in the liver and spleen. The probiotic- and antibiotic-treated groups showed significantly decreased total antioxidant capacity compared to the control group. Spleen is an immune organ and helps in monitoring the body's immune response (41). The spleen and liver are primarily targeting organs for *Salmonella* infection and the pathological state is mainly a cause of oxidative stress. Thus, it is essential to augment the antioxidant defense system in the body during stress as the liver and spleen are the primary immune organs. This destroys the redox balance and brings the development of similar changes in other organs. Excessive levels of proinflammatory cytokines are mainly associated with the generation of reactive oxygen species and excess reactive oxygen species will also stimulate the proinflammatory cytokines, resulting in a cascade of immunological and oxidative stress events (42, 43). The liver is the main metabolic organ and detoxification site of the body. It plays a vital role in the body's defense and its toxic products against bacteria (44).

The relative expression levels of proinflammatory cytokines (IL-6, IL-8, IL-17A, and iNOS) in the liver and spleen were markedly increased in the challenged group (INF) compared to the other treated groups because Ajwain extract (AJE) supplementation significantly alleviated the inhibitory effect of *Salmonella* infection. Our studies were in accordance with the previous studies in which the proinflammatory cytokine (IL-6 and IL-8) levels were downregulated in the Ajwain extract-treated (AJE) group compared to the other groups (45, 46). The expression in chicks treated with Ajwain extract (AJE) and COM (probiotic and Ajwain extract) was significantly downregulated compared to the other groups. The combined treatment (Ajwain extract + Probiotic) induced significant changes in IL-6, IL-8, IL-17A, and iNOS compared to AJE and PRO alone. Proinflammatory factors are released during an immune response resulting in inflammation, which activates downstream signaling pathways such as nuclear factor-κB (NF-κB) and mitogen-activated protein kinase (MAPK) pathway. Lipopolysaccharides of Gram-negative bacteria such as *Salmonella* and *E. coli* can be detected by Toll-like receptor 4 (TLR4), the stimulation of which triggers other signaling pathways leading to the sharp increase in synthesis and release of proinflammatory cytokines, resulting in tissue damage along with high consumption of nutrients (47, 48). The initial activation of the adaptor protein myeloid differentiation factor 88 (MyD88) activates nuclear factor-κB (NF-κB) and mitogen-activated protein kinase (MAPK) pathways. MyD88 activates IL-1R associated kinase-4 (IRAK4), which activates other IRAK family members, TRIF, TIRAP which act as essential messengers to activate downstream kinases, transforming growth factor-β (TGF-β)-activated kinase 1 (TAK1) complexes with TAK1-binding proteins, which then activates the IκB kinase (IKK) complex and MAPK pathway. The IKK complex phosphorylates and ubiquitinates the NF-κB inhibitor IκBα, marking it for degradation. NF-κB is released from IκBα and translocates to the nucleus to initiate the expression of proinflammatory cytokines (49, 50). The MAPK pathway activates the transcription factor activator protein-1 (AP-1), which is also responsible for expressing the proinflammatory cytokines such as IL-6, IL-8, IL-17A, and iNOS.

The chemical–gene interaction network identifies small molecules which interact with the genes of interest. The critical molecules identified in the study were asbestos, PDTC, aspirin, simvastatin, titanium oxide, LPS, LP, methotrexate, arsenic, nicotine, and nickel chloride. Asbestos exposure has been correlated with cirrhosis, hyaline splenic, and hepatic plaques (51). Pyrrolidine dithiocarbamate (PDTC) has been reported to inhibit the development of liver cirrhosis, which is associated with decreased oxidative stress and hepatic necroinflammation (52). In a study on TAA-induced cirrhosis, aspirin was reported to reduce fibrogenesis in rat liver (53). Simvastatin, when given prophylactically, has hepatoprotective actions that prevent endothelial dysfunction and inflammation (54). Recent studies have reported the potential of titanium oxide to generate ROS that induces oxidative stress and inflammation in the liver and intestinal tissue (55). Resveratrol is a naturally occurring polyphenol that significantly decreases oxidative stress in hepatic inflammation (56). Intraperitoneal application of lipopolysaccharide (LPS) alone or other hepatotoxins has induced systemic and hepatic inflammation in rodents (57). Latex protein (LP) fraction has been shown to protect animals from septic shock by modulating nitric oxide and cytokine levels (58). Methotrexate has been associated with fibrosis and fatal hepatic necrosis with various liver-related adverse events ranging from asymptomatic and transaminase elevations (59). Arsenic-induced liver fibrosis induces swelling, degeneration, necrosis of hepatocytes, and inflammatory cell infiltration (60). Nicotine administration has been reported to exert several adverse physiological effects on the liver, including significantly reducing the liver weight and increasing the blood serum nitric oxide level (61). Nickel chloride significantly increases hepatic apoptosis, cell cycle arrest, and inflammatory response in the liver of broiler chickens by activating the NF-κB pathway (62).

A gene disease interaction network was constructed to predict the association between genes and diseases. CXCL8, NOS2, and IL-6 reportedly play a role in inflammation, hyperalgesia, cholestasis, and neoplastic cell transformation. Therefore, understanding the role of genes in disease is of high importance. Therefore, a computational approach to predict genes related to diseases will contribute to the field of disease treatment discovery.

Elucidation of an interactive network between gene chemicals and gene diseases is crucial for discovering novel drugs in drug development and repositioning existing drugs to novel therapeutic targets. The biological network-based approaches are effective in predicting chemical–gene networks. This study identifies potential chemical–gene interactions between IL-6, IL-17A, CXCL8, and NOS2 genes.

## Conclusion

In conclusion, this study showed that the combined effect of Ajwain extract and probiotic has a significant effect on preventing *S. typhimurium* infection in broilers, which leads to new feed formula for the poultry industry for enhancing the health of birds. Addition of both the Ajwain extract and probiotics to the diet of broilers infected with *S. typhimurium* modulated the inflammatory responses. The addition of Ajwain extract in broiler feed has a beneficial effect on the immunity parameters, improved antioxidative status, and lowered bacterial count in the liver and spleen. These results indicate that Ajwain is an excellent dietary additive to poultry feed and can act as a potential antibiotic substitute. This study identifies potential chemical–gene interactions between IL-6, IL-17A, CXCL8, and NOS2 genes. The predicted chemical–gene network will have a significant impact in the discovery of novel therapeutic targets in poultry infection.

## Data Availability Statement

The original contributions presented in the study are included in the article/Supplementary Material, further inquiries can be directed to the corresponding authors.

## Ethics Statement

The animal study was reviewed and approved by Institutional Animal Ethics Committee of SKUAST-Kashmir, India.

## Author Contributions

ZH performed animal experiments. SA designed the study and overall supervised the experiments. IB, MD, and AS wrote the manuscript and performed the expression studies. BB performed the data analysis. AK, MY, SM, and AR helped in data interpretation and analysis. MH and RS helped in experimental work. MD and BB performed proofreading of the manuscript and drug analysis. All authors revised and approved the final version of the manuscript.

## Funding

The Science and Engineering Research Board, Department of Science and Technology, Government of India is duly acknowledged for supporting this study under grant EMR/2017/000580.

## Conflict of Interest

The authors declare that the research was conducted in the absence of any commercial or financial relationships that could be construed as a potential conflict of interest.

## Publisher's Note

All claims expressed in this article are solely those of the authors and do not necessarily represent those of their affiliated organizations, or those of the publisher, the editors and the reviewers. Any product that may be evaluated in this article, or claim that may be made by its manufacturer, is not guaranteed or endorsed by the publisher.
