# Supplementary material for: Pathogenesis-Related Gene Expression in Response to Trachyspermum ammi Supplementation Along With Probiotics in Chicken Salmonellosis and Insights in Drug Therapeutics
Source: Front Vet Sci. 2022 Jun 2;9:866614. doi: 10.3389/fvets.2022.866614 (PMC9201639; doi:10.3389/fvets.2022.866614)
Supplement: Supplementary file 1 [file Table_1.DOCX]

**1. Percentage inhibition of DPPH**

At 700ug/ml the ethanolic extract has shown 77.26% of inhibition when compared with the standard ascorbic acid which has shown 95.75% of inhibition. The inhibition has increased with increase in the concentration of extract.

| **Concentration(µl)** | **Ethanol (% inhibition)** | **Ascorbic Acid (% inhibition)** |
| --- | --- | --- |
| 100 | 23.45 | 33.29 |
| 200 | 29.36 | 49.23 |
| 300 | 35.56 | 54.25 |
| 400 | 40.26 | 66.33 |
| 500 | 52.36 | 72.25 |
| 600 | 62.56 | 85.36 |
| 700 | 77.26 | 95.75 |

**2. FRAP (mmol/g of sample)**

| **Concentration(µl)** | **Ethanol** |
| --- | --- |
| 100 | 1.26±0.23 |
| 200 | 1.46±0.21 |
| 300 | 1.52±0.30 |
| 400 | 1.78±0.26 |
| 500 | 2.33±0.15 |
| 600 | 2.56±0.27 |
| 700 | 3.16±0.22 |
